# Dimensional stability and mechanical properties of extruded-compression biopolymer composites made from selected Nigerian grown wood species at varying proportions

**DOI:** 10.1038/s41598-022-14691-z

**Published:** 2022-06-22

**Authors:** A. O. Oladimeji, F. Z. Agboola, D. O. Oguntayo, K. S. Aina

**Affiliations:** 1 Natural Products Research Laboratory, Industrial Chemistry Unit, Department of Chemical Sciences, Olusegun Agagu University of Science and Technology, Okitipupa, Ondo State Nigeria; 2 Department of Environmental Science and Natural Resources, National Open University of Nigeria, Ibadan Study Center, Ijokodo, Ibadan, Oyo State Nigeria; 3 Department of Civil Engineering, Landmark University, Omu-Aran, Nigeria; 4 Department of Forest Products Development and Utilization, Forestry Research Institute of Nigeria, Forest hill, Jericho, P.M.B 5054, Ibadan, Oyo State Nigeria

**Keywords:** Civil engineering, Materials science

## Abstract

250 µm particle size of wood and polyethylene (PE) materials were compounded at mixing proportions of 60/40, 70/30, and 80/20 (with an increase in polymer to decrease in wood content) and extruded using a single screw extruder at a temperature range of 110–135 °C. The particles of *Gmelina Arborea, Tectona grandis*, *Cordia* *milleni,* and *Nauclea diderichii* with recycled Polyethylene were compounded and compressed at 175 N/mm to produce biopolymer composites. The biopolymer composites were subjected to dimensional stability test at 24 h of the water soak method and the ability to withstand load-bearing capacity was investigated. The outcome of the results shows that extruded-compressive biopolymer composites had values ranging from 0.06–1.43 g/cm^3^, 0.38–3.41%, and 0.82–6.85% for observed density, water absorption, and thickness swelling at 24 h of a water soak test. The mechanical properties values ranged from 0.28 Nmm^−2^–21.35 Nmm^−2^ and 0.44–550.06 Nmm^−2^ for flexural modulus and strength; and 191.43 Nmm^−2^–1857.24 Nmm^−2^ and 0.35 Nmm^−2^–243.75 Nmm^−2^ for tensile modulus and strength respectively. It was observed that moisture uptake and strength displayed by the composites vary accordingly in values obtained for wood species at different mixing proportions. As observed that the more polyethylene content is compounded to wood, the better its dimensional stability, and flexural and tensile properties. The wood particles of *Cordia* *milleni* compounded at a proportion of 60 to 40 (polyethylene/wood) performed best in dimensional stability and load-bearing capacity. This study confirmed the effect of methods on wood species and recycled PE for manufacturing wood polymer-based composite for both indoor and outdoor applications.

## Introduction

Since the beginning of the twentieth century, the polymer sector has been expanding; various resin producers and chemical firms throughout the world are primarily contributing to the volume of plastic products produced annually, which exceeds 200 million tons^1,2^. This allows polymer processing industry around the world to be expanding from tens of thousands of small and medium sized enterprises. Most polymer manufacturers use different machines for operation; most use extruders and injection molding machines. The first operation of polymer production is through the pelletizing die while the second is for the final shaping (Vlachopoulos and Wagner, 2001). The two operations involve heating and melting of polymer, by pumping the melted polymer to the shaping unit to form the required shape and dimensions, after cooling to solidify. The compounding of polymer and other particles like wood is typically done using screw extruders under specific heat and pressure. The compounded material can be pressed or shaped into an end product or formed into pellets for further processing in an injection moulding machine. The polymer products can be manufactured in sheet or profile extrusion, injection moulding, calendering, thermoforming, or compression moulding^4^.

The polymer products have unique properties that include easy fabrication, low densities, resistance to corrosion, electrical and thermal insulation, and often favorable rigidity and toughness per unit weight^3^. Due to these properties displayed, the polymer industry has continued to grow in developing countries where their needs in transportation, food packaging, housing, and electrical appliances are very paramount. The interest in adding wood fiber as reinforcement to polymer has grown over the years due to the outstanding properties and performance of the products^5^. Wood polymer composite has been known to be an alternative bioproduct to organic bonded particleboards with improved characteristics to suit different applications^4^. The combination of wood and polymer has shown mechanically improved products compared to other wood-based panel products and plastic products^6^. Direct extrusion is the most common technique used in the manufacture of biopolymer composites. This technique allows the raw materials to be melt-compounded and extruded into a continuous profile by forcing the molten material through the die in the same process step^7^. The Indirect extrusion technique can either be in profiles or sheet materials for compression moulding to be manufactured. This study adopts both techniques for the production of biopolymer from selected wood species grown in Nigeria; it was aimed to investigate their effect on specific properties like mechanical and dimensional stability of the product. Many particles of wood species from temperate and tropical regions have been investigated. Species such as pinus, maple, and oak are commonly used for the production of commercial wood-plastic composites products in the temperate region^8^. Previous research has shown that wood species affect the mechanical properties of WPCs, with hardwood particles outperforming softwood flours in terms of tensile properties and heat deflection temperature^5,9^. Most wood-plastic composites manufacturers are found in developed countries of the world with improved and advanced technologies, as the technology is improving and marketing demand is also growing. As the industry is growing in developed countries, the developing countries are still finding it difficult to align themselves to the technologies despite their huge generation of wood waste from the numerous wood mill industries^10,11^. The wood waste generated by the wood mill industries could be put to important industrial use for the production of WPCs rather than being used in landfills or burnt^12^. There is a gradual increase in the trend of research on WPCs in Nigeria with the use of different plastic binders and wood species, being assessed. The effect of some tropical wood species on the strength properties of WPCs has also been investigated^13,14^. Investigated the possibility and potential of tropical wood species and agro-residue for the production of WPCs in Nigeria, using a screw extruder and manually fabricated compounding hot press machine. It is worth noting that Nigerian grown hardwood species such as *Ceiba pentandra*, *Triplochiton scleroxylon*, *Entandrophragma cylindricum*, *Cordia alliodora*, *Funtumia Elastica*, *Brachystegia Kennedy*, *khaya ivorensis*, *Tectona grandis, Terminalia Superba* and *Milicia excelsa* have been used to produce WPCs without coupling agents using a single screw extruder and/or compression molded^5,15–18^. These studies revealed improved strength products with low sorption properties that can be used for low-stress indoor applications^17^. All these wood species are regularly found in the daily wood conversion process in Nigerian wood milling industries for structural purposes. Recently, as research on WPCs is growing, more and more Nigerian grown wood species are also required to be investigated. Among the wood species previously investigated are *Gmelina Arborea and Tectona grandis* which falls to the specific gravities of 0.42–0.64 and 0.61–0.73. these wood species are common to Nigerian sawmills due to high demand market values for export, the wood specie is useful for paper making, molding furniture, interior woodworking, shipbuilding and plywood, pole wood, particleboards, veneer, and some other structural^19,20^. These wood species are used in this study to compare with the new species like *Cordia milleni* and *Nauclea diderichii* that are yet to be investigated. These wood species are semi-deciduous forest wood with specific gravities of 0.41–0.50 and 0.56–0.63 for *Cordia milleni* and *Nauclea diderichii*. Both woods species are found to have lower specific gravities than Gmelina Arborea and Tectona grandis, they also have good characteristics that made them useful for general construction and wood-based panel products; specifically, *Nauclea diderichii* is found to be very useful for outdoor purposes such as railway sleepers, heavy construction, hydraulic works in contact with fresh or seawater^21,22^. The use of wood particles in the plastic industry is projected to increase as the demand for WPCs products in the construction industry is gradually growing for roofing, tiles, and window frames^5,23^. Commercial applications of WPCs are very high for decking and siding which seem to be evidence of future economic development and growth for developed countries^4,24^. WPCs are gradually extending their popularity to developing countries like Nigeria and the need for targeted commercialization to enhance structural applications in developing countries requires intensive research in both materials and technology.

The present challenge of WPCs research and development in Nigeria can be related to the availability of machineries for milling, processing, and production. Despite having the same techniques as the polymer industry, most manufacturers of polymer composites find it difficult to allow their machines to be used for WPCs research. Most polymer products are used for packaging and domestic uses, the rate of consumption is high, and used are daily and regularly found littering the streets^25^. Studies have shown that recycled plastics can be used^26,27^. In Western Europe, almost 40% of all recycled plastic goods were utilized in distribution products like film and bags, while 30% were used in building applications like pipes, windows, and tiles^28^. Recycled plastics have huge potential for producing WPCs at lower costs with better qualities than virgin plastics. The sawdust from sawmills and polymer wastes are seriously environmental pollutants that need urgent attention in most developing countries of the world. Recycling plastic and sawdust will serve as possible raw materials for the production of biopolymer composites which can serve as a solution to combat environmental pollution and avert disasters as well.

As a result of this environmental challenge, this study seeks to provide information on the dimensional stability and mechanical properties of extruded-compressed WPCs reinforced with wood particles from four Nigerian grown wood species. This study considered factors such as specific particle size, processing temperature, and pressure as a constant against variables factors such as plastic/wood ratio and different indigenous wood species on a single screw extruder machine fabricated locally in Nigeria.

## Materials and methods

The wood species used to make the samples in this investigation came from a variety of sources, which include *Gmelina Arborea, Tectona grandis*, *Cordia* *milleni*, and *Nauclea diderichii*. The sawmill branch of the Department of Forest Products Development and Utilization at the Forestry Research Institute of Nigeria, Ibadan, Oyo State, collected wood particles from these wood species. The waste plastic packaged water bags were supplied by the water packaging factory of DFRIN Consultancy Company at Forestry Research Institute of Nigeria. All the methods were performed in accordance with relevant guidelines and regulations.

### Manufacture of the experimental samples

**Figure 1:**
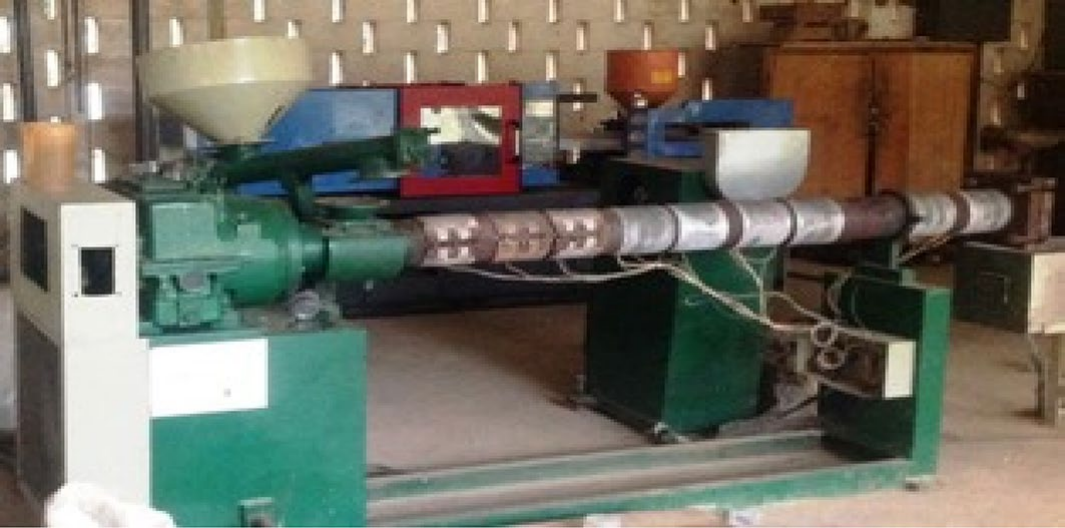
Single screw extruder.

**Figure 2:**
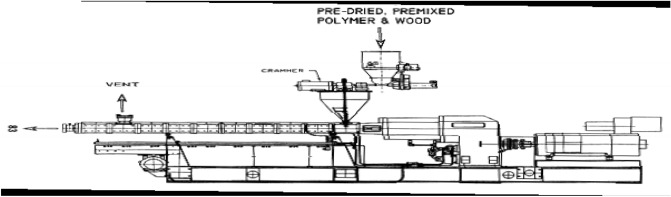
Premix extruder.

Fresh wood particles derived from sawn wood of *Gmelina Arborea; Tectona grandis*, *Cordia* *milleni*, and *Nauclea diderichii* were thoroughly sieved with wire mesh of size 0.25 mm (250 µm) to obtain wood dust. Each wood species' homogeneously screened wood dust was oven-dried at 103 ± 2 °C for 24 h to attain 4% moisture content. The packaged water bags were thoroughly washed to remove stains and unwanted particles like sand. It was dried and milled into particles using an industrial hammer mill machine of 50 hp (manufactured by Lucas engineering company, Lagos, Nigeria) available at the Department of Forest Products Development and Utilization and thoroughly sieved with wire mesh of size 0.25 mm (250 µm). Each proportion of wood and recycled PE required for samples were thoroughly hand mixed and fed into a single screw extruder (manufactured by Lucas engineering company, Lagos, Nigeria), also available at the Department of Forest Products Development and Utilization (Fig. 1). The extruder has a hopper for feeding materials to the machine; these materials were premixed and allowed to travel through the barrel at a controlled temperature provided by the heaters; the barrel has a screw designed to mix, blend and push the molten material out through the die (Fig. 2). The molten materials were injected into a metal mould of dimensional size 6 cm × 6 cm × 12 cm and compressed to produce flat platen boards under a hydraulic press machine of 175 N/mm for 45 min. After that, the boards were stripped open from the mould and cut into test specimens according to^29,30^ to determine dimensional and mechanical properties.

### Determination of properties

#### Density

Before the water immersion test, the density of the composite was calculated using the oven-dry weight and volume. Density is the ratio of the mass of a test piece to its volume, calculated to the nearest 0.01 g/cm^3^ in accordance with^31^, and calculated with D = m/v, where D is the density in (g/cm^3^), m is the mass in (g), and V is the volume in (cm^3^). The test pieces were square with sides measuring 100 mm. the specimen was conditioned to a constant mass in an atmosphere of relative humidity of 65 ± 5% and a temperature of 20 °C ± 2 °C.

#### Dimensional tests

Specimen size of 76.2 mm × 25.4 mm × 6.4 mm in each WPC was measured and subjected to water immersion treatment for 24 h in accordance with^29^, at a room temperature of 26 ± 1 °C, this is to observe the reaction of the WPC samples to water exposure. The measurements such as weight, length, and thickness of each sample were taken after removing from water at a stipulated period and all surface water had been wiped off with a dry cloth. These tests were calculated using Water absorption (%) = W_t_−W_o_/W_o _× 100%, where W_o_ and W_t_ are the oven-dry mass (g) and the mass (g) after time t, in the water immersion test, respectively. The thickness of each composite sample was measured during the water immersion testing to determine the thickness swelling (TS) using the following equation: Thickness swelling (%) = T_t_−T_o_/T_o_ × 100%, where T_o_ and T_t_ are the panel thickness (mm) before and after the water immersion, respectively.

#### Flexural strength and modulus

Three-point bending tests were performed on flexural strength specimens with dimensions of 123.5 mm × 12.7 mm × 6.4 mm (thickness) using a standard Universal Testing Machine of the WDW model (Jinan Hensgrand Instrument Co., Ltd., Jinan, China). In compliance with^30^, an 858 load frame with a 50 kN load cell and a crosshead speed of 2.8 mm/min was used. The bending tests were also repeated three times for each composite formulation at ambient temperatures of 23 2 °C and 50 5% relative humidity. The flexural strength (MOR) was calculated for the load–deflection curve by using S = 3PL/2bh^2^, where *S *is the maximum bending stress,* P *is the load at a given point on the load–deflection curve, in (N); *L *is support span, in (mm); *b *is the width of beam tested, in (mm).; and *h *is the depth of beam tested, (mm). In accordance with^30^, which described the determination of the modulus of elasticity (MOE) or flexural modulus, which is the ratio of stress to corresponding strain. It is calculated by drawing a tangent to the steepest initial straight-line portion of the load–deflection curve, which is essentially a load at which the specimen deflects by 1 (mm). The flexural modulus of the elasticity (MOE) in the bending tests is calculated within the linear limit by using E = PL^3^/4bh^3^D, where *E *is the modulus of elasticity (flexural modulus) in bending, in (N/mm^2^); *P *is the load at the steepest initial straight-line portion of the load–deflection curve, in (N); *L *is support span, in (mm)*;* *b *is the width of beam tested, in (mm); *h *is the depth of the beam tested, in. (mm); and *D *is deflection at the load *P *in (mm).

#### Tensile strength and modulus

Universal Testing Machine was used to test Type-I tensile bar specimens with dimensions of 165 mm × 19 mm × 6.4 mm (thickness) of model WDW (Jinan Hensgrand Instrument Co., Ltd., Jinan, China) 810 load frame with 50 kN load cell at a crosshead speed of 2.8 mm/min and lower support of 100 mm. An extensometer was used to measure the specimen's elongation (strain) along a 25 mm gauge length. The tensile tests were performed in accordance with^32^. For each composite formulation, all measurements were carried out at ambient temperatures (23 2 °C and 50% RH for at least 40 h) and reproduced five times. The maximum load in Newton was divided by the original minimum cross-sectional area of the specimen in square meters to compute the tensile strength. Young’s modulus of elasticity (MOE) was calculated using the initial linear part from the load-elongation curves. The MOE is calculated by dividing the stress rise throughout this linear period by the strain increase.

### Data analysis

Graphical analysis and analysis of variance were utilized to process the test data gathered for the assessment of the study variables used in this study. The graphical analysis makes it simple to see the trend of any possible relationship between the study variable and a certain board attribute. 3 by 4 factorial designs in completely randomized design were adopted to determine the significance level of the principal and interacting effect that could emerge. This study employed the SPSS (Statistical Package for the Social Sciences) version 20.0 package for analysis of variance (ANOVA). To evaluate the difference between the means and identify the optimal treatment combination from the criteria considered, Duncan's Multiple Range Test (DMRT) was used to separate the treatment means. The ANOVA was used to determine the relative importance of numerous sources of variation in board density, water absorption, thickness swelling, modulus of rupture, and elasticity in flexural and tensile applications. Differences in wood species and proportionate ratio were the main effects studied. The interaction effects between the primary components were also considered. The WPCs were produced at three proportional levels of PE/wood of 60/40, 70/30, and 80/20 at wood species of *Gmelina Arborea*, *Tectona grandis*, *Cordia* *milleni*, and *Naudea diderichii*.

## Results and discussion

### Physical properties

**Figure 3:**
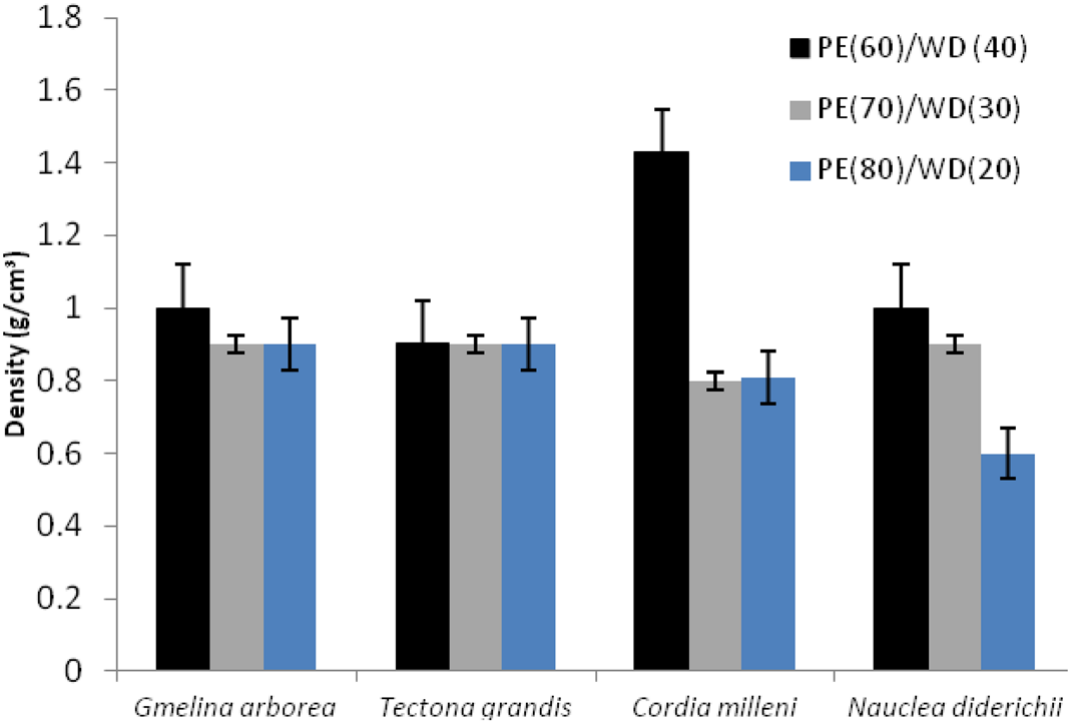
Density observed from extruded-compressed biopolymer composites.

**Figure 4:**
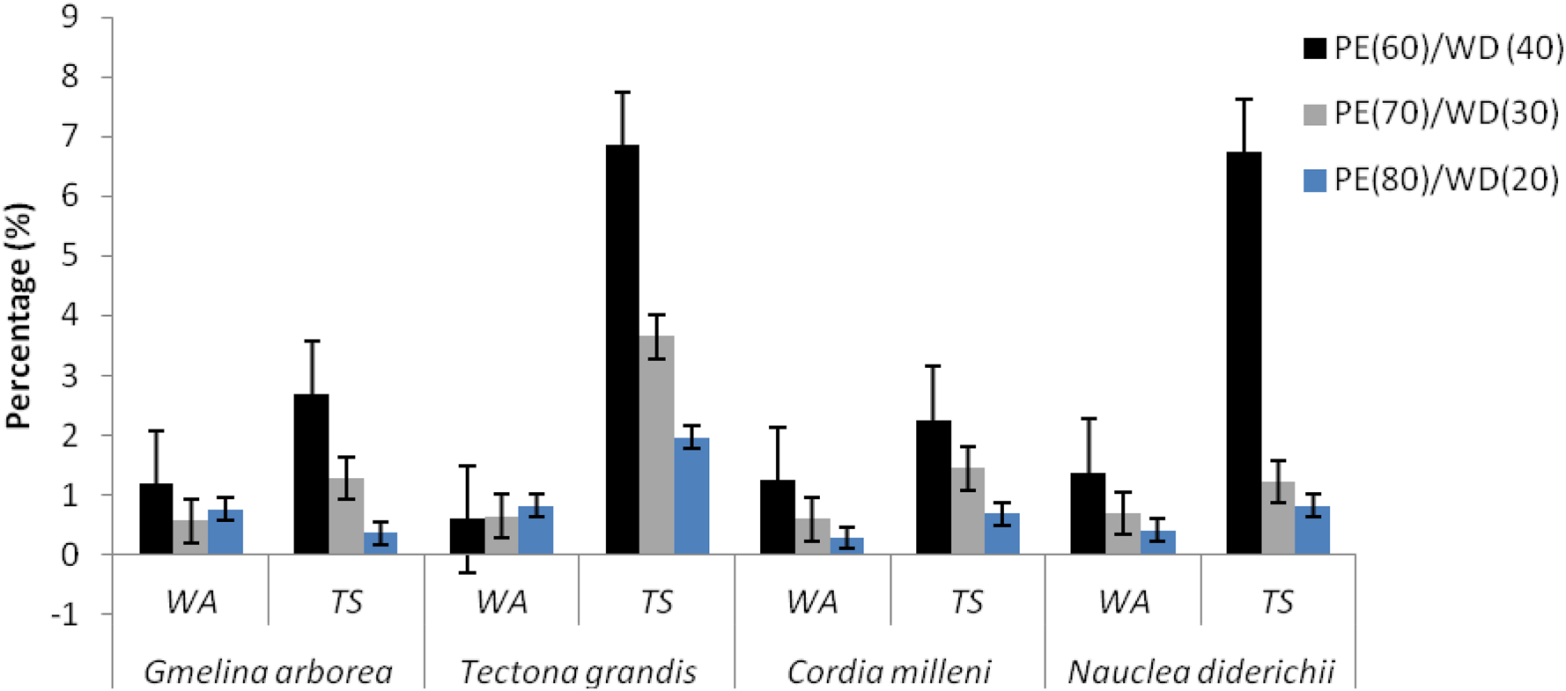
Dimensional stability observed from extruded-compressed biopolymer composites. *WA* water absorption, *TS* thickness swelling, *PE* Polyethylene, *WD* wood.

The mean values for all the properties are presented in Table 1. It shows that the observed density, water absorption, and thickness swelling values obtained in this investigation ranged from 0.06 g/cm^3^ to 1.43 g/cm^3^, 0.38% to 3.41%, and 0.82% to 6.85%, respectively. As presented in Table 1, the density observed for the composites varies according to the different wood species. Similarly, in Table 1, the density observed also varies according to the different mixing proportions. *Cordia* *milleni* had the highest density of the wood species used in the study, compared to the other wood species. In the mixing ratio, 70/30 (PE/wood) has the highest density observed at 0.38 g/cm^3^, followed by 60/40 and 80/20 (PE/wood) at 0.20 g/cm^3^ and 0.20 g/cm^3^. However, in water absorption and thickness swelling, Table 1 shows that water absorption values obtained for each wood species were 0.84%, 1.37%, 1.49%, and 0.68% for *Gmelina Arborea, Cordia milleni, Nauclea diderichii,* and *Tectona grandis,* respectively. The thickness swelling values obtained for each wood species were 1.77%, 2.13%, 2.93%, and 4.82% for *Gmelina Arborea, Cordia milleni, Nauclea diderichii, and Tectona grandis,* respectively (Table 1). Among the mixing proportion employed in the study, the value obtained varied differently for water absorption and thickness swelling and was found to be 0.85%, 0.62%, 1.82%, and 4.28%, 2.25%, 2.20% for mixing proportions 60/40, 70/30, and 80/20 respectively (Table 1). As illustrated in Figs. 3, 4, the entire physical properties show a similar trend for observed density, water absorption, and thickness swelling; the values obtained decrease as the proportion of wood decreases to plastic content. The physical properties observed on the composites showed that observed density, water absorption, and thickness swelling values obtained for each wood species vary differently from each other’s (Figs. 3, 4). The reaction of the composites to the water-soaking test revealed that the boards behave similarly to plastic because it has more plastic as a matrix, which encapsulated the fiber and prevented it from absorbing moisture, thereby making it have high dimensional stability. This implies that as the proportion of plastic increases, the composition's water absorption and thickness swelling values decrease (Figs. 3 and 4). The moisture uptake by the boards made from different wood species also varies with the mixing proportions employed for production. The values obtained in this study agree with other values obtained in previous studies^8,14,26,33^. The outcome also agrees with the observations found in previous studies by^26^; he showed a decrease in dimensional properties of WPC manufactured from particles of *Gmelina* Arborea with high recycled polyethylene. The author also confirmed that increased recycled plastic content in the wood fillers gives better dimensional stability boards. A similar observation was also witnessed in this study, which might be attributed to the inherent interbond relationship between plastic and wood interfaces. The structural interface of plastic with wood filler was found to be high in this study; the pores were limited due to the compressional force exerted on the molten mixture, and the tiny pores were also found to occur at the surfaces of the composites. Plastic found in this composite prevented the naturally hydrophilic wood from absorbing moisture when it gets into contact with water. As earlier reported, the proportion of plastic matters when biopolymer composite products are exposed to moisture contact; more plastic content than wood content reduces water residence sites for water uptake on the composite. The outcome of the analysis of variance for density observed water absorption and thickness swelling are presented in Table 2. As depicted in Table 2, all factors (main and two factors interaction) were significant at a 5% probability level for density observed while not significant in water absorption. In thickness swelling, only the main factors are significant at a 5% level of probability.

**Table 1:** Mean values obtained for physicomechanical properties of extruded-compressed biopolymer composite.

Wood species	Plastic (%)	Wood (%)	Observed density (g/cm^3^)	Water absorption (%) at 24 h	Thickness swelling (%) at 24 h	Mechanical properties (N/mm^2^)			
						Flexural modulus (N/mm^2^)	Flexural strength (N/mm^2^)	Tensile modulus (N/mm^2^)	Tensile strength (N/mm^2^)
*Gmelina Arborea*	60 70 80	40 30 20	1.00 ± 0.01 0.90 ± 0.09 0.90 ± 0.05	1.18 ± 0.02 0.56 ± 0.02 0.76 ± 0.34	2.66 ± 0.28 1.28 ± 0.19 0.36 ± 0.17	224.48 ± 1.85 260.61 ± 1.82 540.89 ± 1.29	20.52 ± 2.60 12.85 ± 2.02 11.01 ± 0.87	1885.72 ± 300.35 1600.03 ± 395.54 1137.58 ± 333.05	24.38 ± 5.97 17.59 ± 2.78 14.65 ± 0.35
*Tectona grandis*	60 70 80	40 30 20	0.90 ± 0.02 0.90 ± 0.01 0.90 ± 0.05	1.57 ± 0.04 0.64 ± 0.04 0.82 ± 0.13	6.85 ± 5.23 3.65 ± 0.32 1.96 ± 2.11	261.53 ± 2.44 285.30 ± 1.24 514.58 ± 2.54	18.85 ± 1.11 11.51 ± 3.47 8.34 ± 0.28	1291.88 ± 366.84 1330.03 ± 234.43 1416.62 ± 438.70	18.56 ± 2.64 17.34 ± 7.96 16.14 ± 4.51
*Cordia milleni*	60 70 80	40 30 20	1.43 ± 0.27 0.80 ± 0.19 0.81 ± 0.23	1.25 ± 0.45 0.59 ± 0.22 0.28 ± 1.49	2.26 ± 0.58 1.45 ± 0.13 0.68 ± 1.22	433.85 ± 2.34 500.98 ± 2.17 550.06 ± 2.70	21.35 ± 2.31 19.51 ± 8.66 18.18 ± 1.55	1015.01 ± 381.79 1509.42 ± 353.42 1687.34 ± 309.28	23.94 ± 9.10 18.96 ± 4.52 11.43 ± 9.79
*Nauclea diderichii*	60 70 80	40 30 20	1.00 ± 0.03 0.90 ± 0.07 0.60 ± 0.03	1.38 ± 0.10 0.69 ± 0.21 0.41 ± 1.34	6.73 ± 4.43 1.22 ± 0.26 0.82 ± 0.28	385.05 ± 2.34 413.56 ± 1.66 452.93 ± 8.44	16.68 ± 2.89 17.17 ± 0.29 13.68 ± 2.89	1059.00 ± 191.43 1200.88 ± 208.87 1560.99 ± 241.86	20.65 ± 9.72 18.96 ± 4.51 17.52 ± 3.89

Each value represents the mean of five replicates with standard deviations.

**Table 2:** Summary of statistical analysis of variance carried out on data obtained for physicomechanical properties.

Variable factors	Density (g/cm^3^)		Water absorption at 24 h (%)		Thickness swelling at 24 h (%)		Flexural strength (N/mm^2^)		Flexural modulus (N/mm^2^)		Tensile strength (N/mm^2^)		Tensile modulus (N/mm^2^)	
	F value	Sig	F value	Sig	F value	Sig	F value	Sig	F value	Sig	F value	Sig	F value	Sig
Wood species	169.106	0.00*	0.800	0.50^ns^	3.731	0.02*	2.334	0.09^ns^	3.288	0.04*	0.827	0.49^ns^	2.760	0.05*
Mixing proportion	7.024	0.00*	2.697	0.08^ns^	3.777	0.03*	3.549	0.04*	3.168	0.06^ns^	7.164	0.00*	2.505	0.10^ns^
Wood sp. x Mixing P	7.019	0.00*	1.112	0.38^ns^	2.090	0.09^ns^	2.385	0.06^ns^	1.657	0.17^ns^	8.343	0.00*	7.171	0.00*

* represents significant, ns represents not significant at 5% level of probability, while sp represents species and P represents proportion.

### Mechanical properties

#### Flexural properties

**Figure 5:**
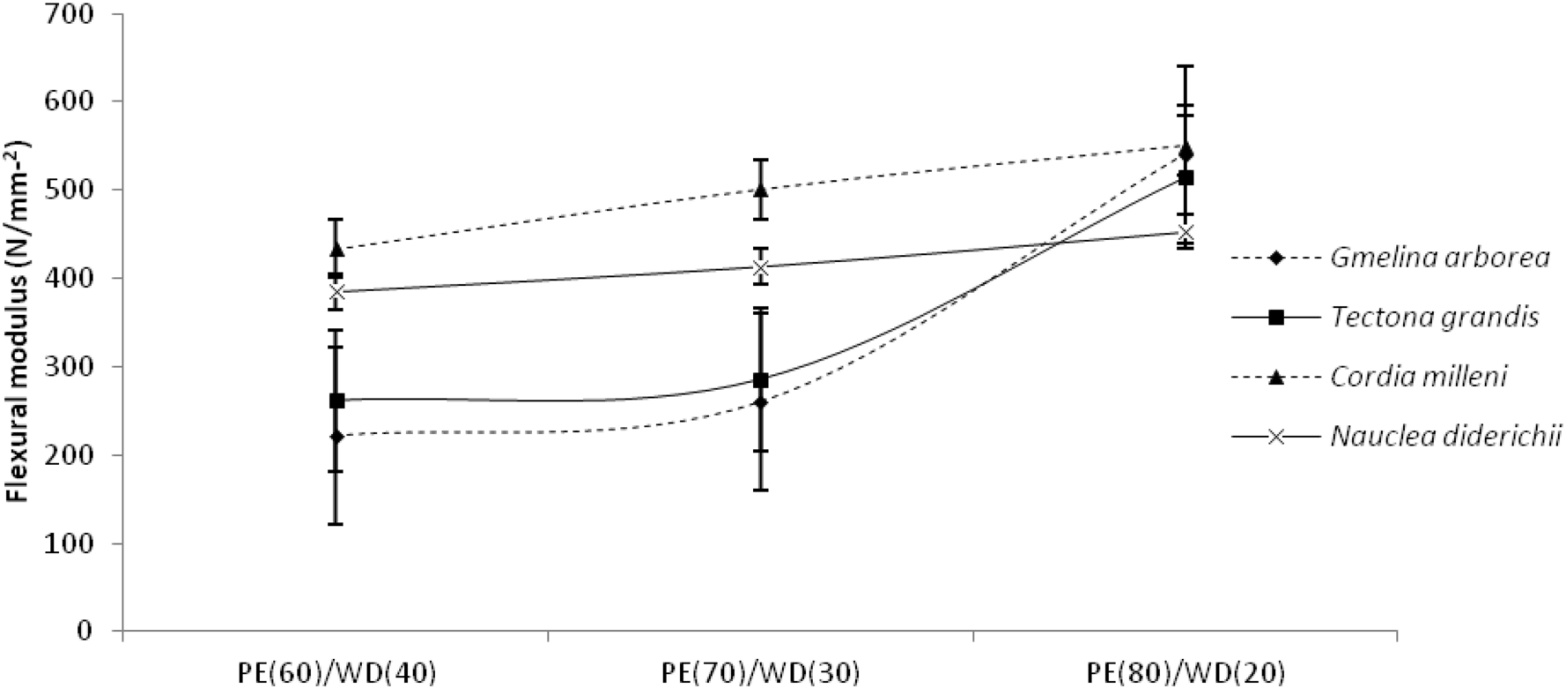
Flexural modulus of extruded-compressed biopolymer composites.

**Figure 6:**
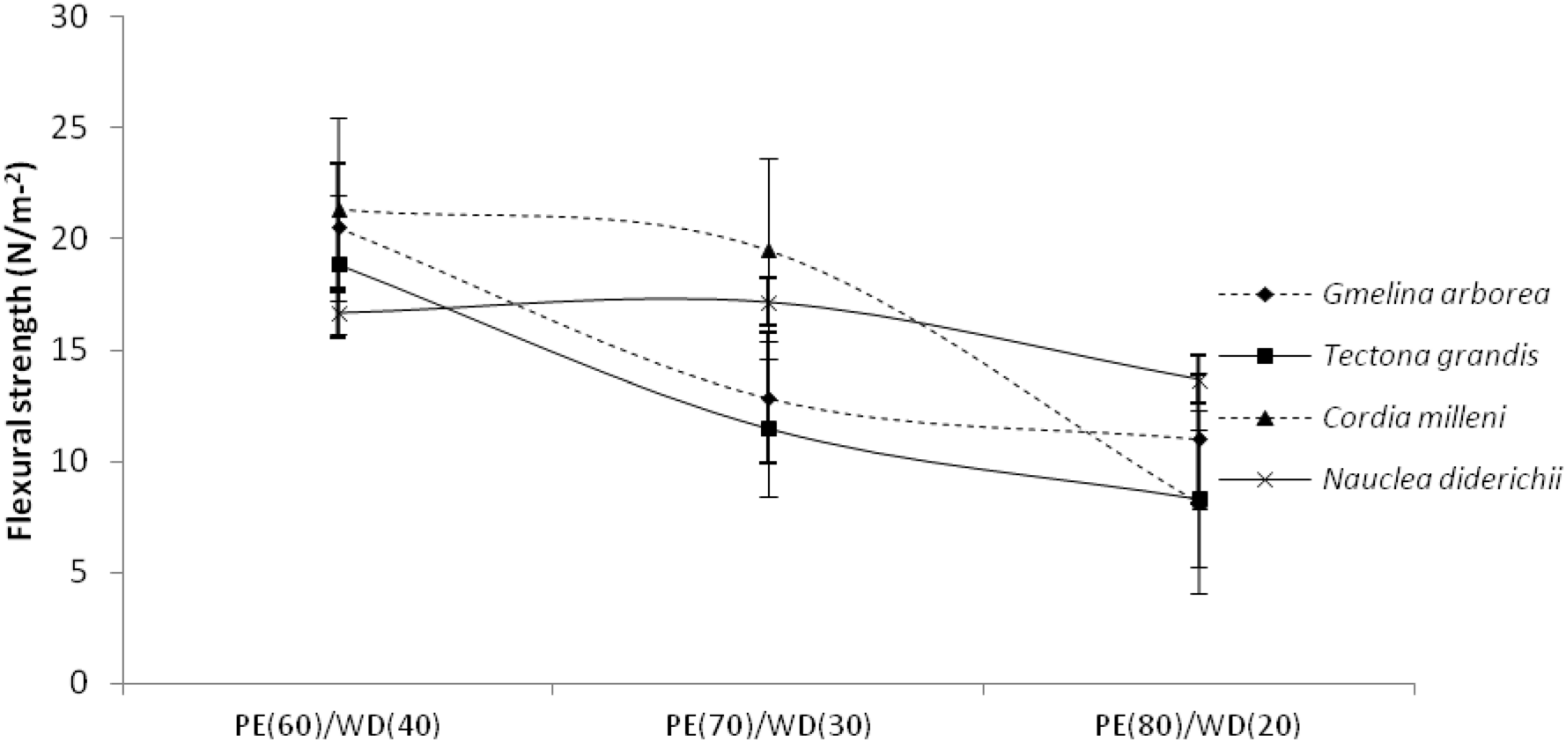
Flexural strength of extruded-compressed biopolymer composites.

Table 1 shows the mean flexural characteristics of biopolymer composites generated from various wood species and mixing fractions. As presented in Table 3, the values obtained for flexural strength and modulus ranged from 8.34 Nmm^−2^ to 21.35 Nmm^−2^ and 185.05 Nmm^−2^ to 550.8 Nmm^−2^, respectively. As presented in Table 1, the flexural strength and modulus values obtained among the wood species were 14.79 Nmm^−2^, 19.69 Nmm^−2^, 12.52 Nmm^−2^, 12.90 Nmm^−2^, 341.99 Nmm^−2^, 494.97 Nmm^−2^, 350.52 Nmm^−2^, and 353.81 Nmm^−2^ for *Gmelina Arborea, Cordia milleni, Tectona grandis* and *Nauclea diderichii* respectively. While between the mixing proportion, the flexural modulus and strength obtained were 17.52 Nmm^−2^, 11.97 Nmm^−2^, 15.43 Nmm^−2^, and 457.30 Nmm^−2^, 302.04 Nmm^−2^, 396.62 Nmm^−2^ for 60/40, 70/30, 80/20, respectively (Table 1). As illustrated in Figs. 5, 6, the flexural modulus and strength face the same trend. As the proportion of wood fibers decreases, the flexural values obtained also decrease.

**Table 3:** Result of duncan multiple range test carried out on the outcome of ANOVA results for physical and mechanical properties.

Variables	Levels	Physical properties			Mechanical properties (N/mm^2^)			
		Density (g/cm^3^)	Water absorption (%)	Thickness swelling (%)	Flexural Strength	Flexural Modulus	Tensile Strength	Tensile Modulus
Wood species	*Gmelina .arborea* *Cordia milleni* *Nauclea diderichii* *Tectona grandis*	1.008^ab^ 1.012^a^ 1.001^b^ 1.001^b^	0.835^a^ 1.373^a^ 1.492^a^ 0.675^a^	1.767^a^ 2.130^ab^ 2.932^b^ 2.820^b^	341.99^a^ 494.97^a^ 353.81^a^ 350.52^a^	14.79^ab^ 19.69^a^ 12.90^b^ 12.52^b^	18.83^a^ 18.84^a^ 17.35^a^ 19.04^a^	1541.10^a^ 1404.21^b^ 1346.75^c^ 1273.25^d^
Mixing proportion (PE/wood)	60/40 70/30 80/20	0.204^b^ 0.359^a^ 0.103^c^	0.845^a^ 0.620^a^ 0.416^a^	4.282^b^ 2.251^a^ 2.204^a^	457.29^a^ 302.04^b^ 396.62^ab^	17.52^a^ 11.97^a^ 15.43^a^	20.79^a^ 17.02^b^ 17.73^b^	1481.87^a^ 1398.59^a^ 1294.76^a^

The same letter represents no significance while different letter represents significance to one another.

Furthermore, the flexural strength and modulus found in the biopolymer composites vary differently in each wood species (Figs. 5, 6). Table 2 shows the output of the flexural properties analysis of variance data. As presented in Table 2, only the wood species factor was significant for flexural modulus, while the mixing proportion factor was also significant at a 5% probability level for flexural strength. The outcome of these results implies that wood species influence the flexural modulus of biopolymer composites while the mixing proportion of PE/wood also influences the strength properties of biopolymer composites. Table 3 shows the results of the Duncan multiple range test (DMRT) conducted at the level of significance; the results were presented in mean values with alphabetical letters showing the level of significance among and between the main factors considered in this study, biopolymer composites made from particles of *Nauclea diderichii, Tectona grandis,* and *Gmelina Arborea* were not different to each other in flexural modulus and strength but significant to biopolymer composites made from *Cordia milleni*. The followed-up test also confirmed that biopolymer composites made from 70/30 and 80/20 were not significant to each other but significant to those made from 60/40 (PE/wood).

#### Tensile properties

Table 1 shows the mean values of tensile characteristics obtained in this investigation for biopolymer composites manufactured from several wood species at various mixing ratios. As presented in Table 1, the values obtained for tensile strength and modulus ranged from 1015.00 Nmm^−2^ to 1885.72 Nmm^−2^ and.

**Figure 7:**
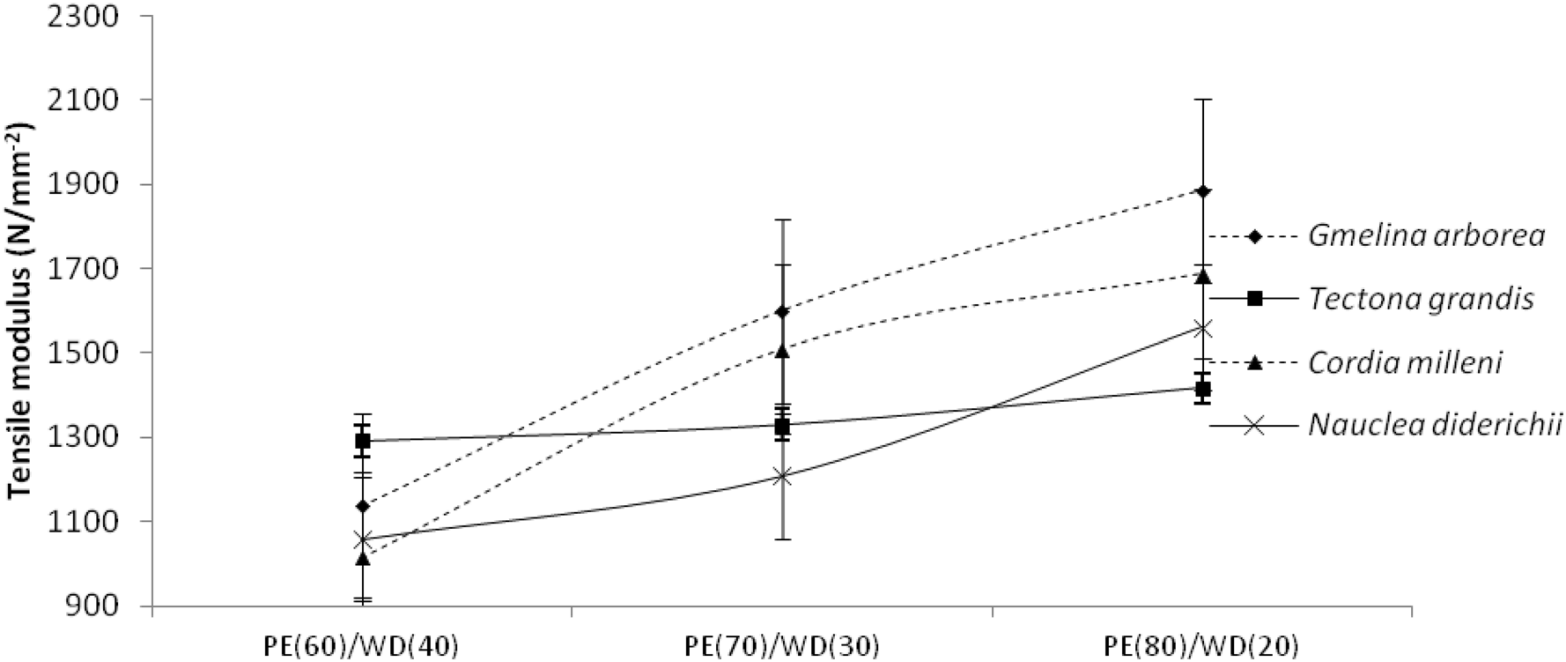
Tensile modulus of extruded-compressed biopolymer composites.

**Figure 8:**
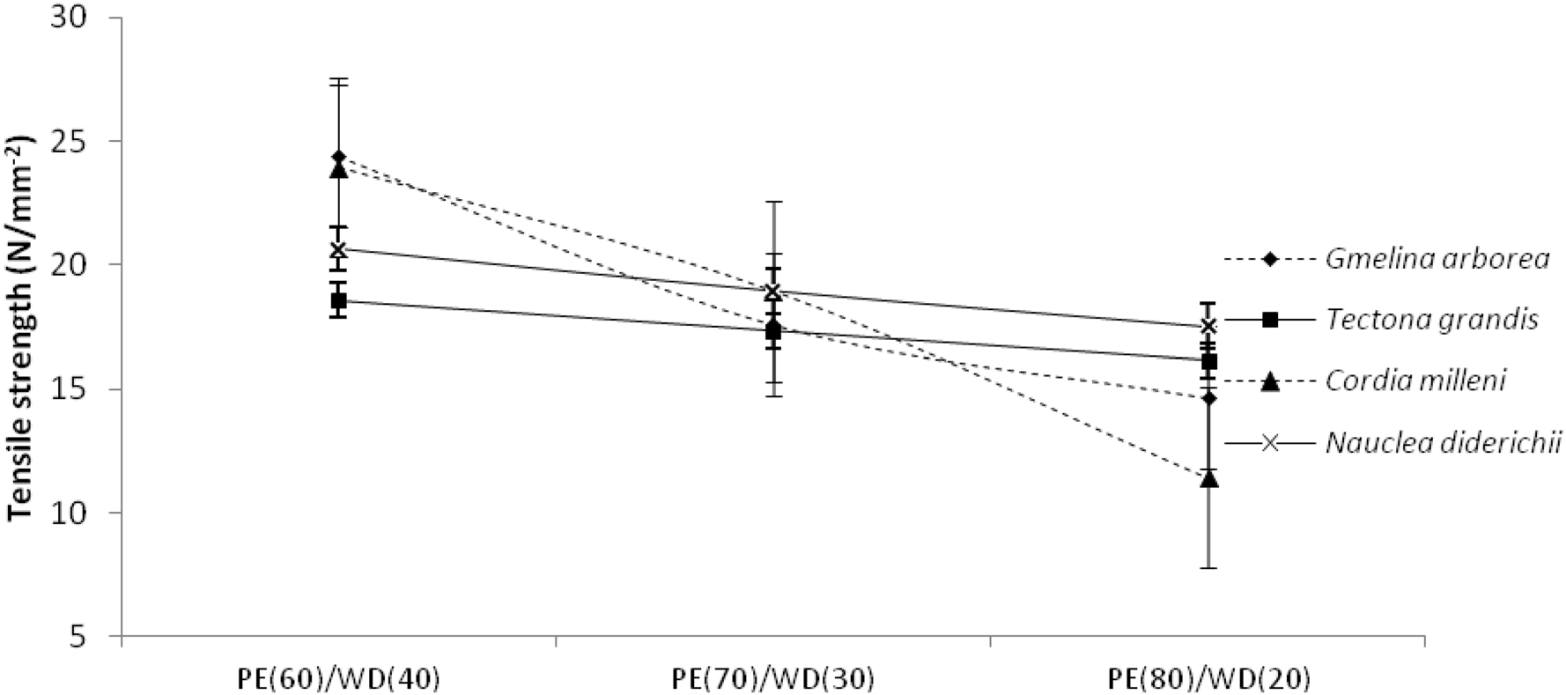
Tensile strength of extruded-compressed biopolymer composites.

11.43 Nmm^−2^ to 24.38 Nmm^−2^ respectively. As presented in Table 1, the tensile modulus and strength values obtained among the wood species were 18.83 Nmm^−2^, 18.84 Nmm^−2^, 19.04 Nmm^−2^, 17.35 Nmm^−2^ and 1541.01 Nmm^−2^, 1404.21 Nmm^−2^, 1273.25 Nmm^−2^, 1346.75 Nmm^−2^ for *Gmelina Arborea, Cordia milleni, Nauclea diderichii, *and *Tectona grandis* respectively. At the same time, the values obtained for the mixing proportions for tensile modulus and strength were 1481.87 Nmm^−2^, 1398.59 Nmm^−2^, 1294.76 Nmm^−2^, and 20.79 Nmm^−2^, 17.02 Nmm^−2^ 17.73 Nmm^−2^ for 60/40, 70/30, 80/20 respectively (Table 3). As illustrated in Fig. 7 and 8, the tensile modulus and strength had the same trend; as the proportion of wood decreases, the values obtained for tensile decreases.

Furthermore, the tensile strength and modulus values obtained vary depending on the wood species (as seen in Figs. 7 and 8). As depicted in Table 2, at a 5% level of probability, the analysis of variance demonstrates that both wood species and mixing fraction are significant. The followed-up test shows that tensile modulus for biopolymer composites made from *Nauclea diderichii, Tectona grandis,* and *Cordia milleni* was not significant to each other but significant to *Gmelina Arborea*. In mixing proportion, biopolymer composites of 80/20, and 70/30 were not significant to each other but significant to 60/40 (PE/wood). Contrary to the report for modulus, strength was different; all biopolymer composites made from *Gmelina Arborea, Nauclea diderichii, Tectona grandis,* and *Cordia milleni* were not significant to each other. But the same pattern with the modulus was seen in mixing proportion for strength, and biopolymer composites were 70/30 and 80/20, not significant to each other but significant at 60/40 (Table 3). This study observed that as the proportion of plastic to wood increases, the tensile and flexural properties decrease (Figs. 5, 6, 7, 8).

The flexural and tensile modulus values obtained in this study agree with the study conducted by^34,35^. As seen in Table 3, the values obtained by the biopolymer composites for strength properties could be attributed to the incompatibility between the particles of wood used and the polymer matrix. It has been noted that interfacial adhesion between the two-component materials gives better mechanical properties to the composite^36^. It could also be attributed to inter-structural bonding fiber to fiber arrangement (interwoven), which overlapped to increase stress concentration, leading to higher strength^12^ also reported that the lower MOE of the composites could be mainly attributed to the insufficient interfacial interaction between polymeric matrix and wood particles, which may not allow efficient stress transfer between the two phases of material which depend on the proportion used. There was variation in the strength and modulus of composites made from different wood species. This could be due to the interaction of each wood species' strength or density with the polymer, which determined the composites' mechanical properties. In a study conducted by^34^, it was discovered that hardwood particles outperform softwood flour in terms of tensile characteristics and heat deflection temperature (the temperature at which a polymer or plastic sample deforms under a particular load). It however means that some wood species particles will have higher mechanical property enhancements than other species. It was observed in this study that *Cordia* *Milleni and Gmelina Arborea particles* performed better in flexural and tensile properties than other wood species. This study further observed that as the plastic content increased to wood content, composites made with *Tectonia Grandis* and *Nauclea diderichii* have higher tensile properties than the lower density wood species like *Cordia* *milleni* and *Gmelina Arborea* (Figs. 7 and 8). This observation collaborated with the report by^34^ but was seen to be accurate with the proportion of 80/20 of plastic content to wood content. This observation was different in flexural properties for biopolymer composites, as seen in Figs. 5 and 6, lower density wood species particles (*Cordia* *milleni* maintained the lead in flexural properties than others at all the proportional ratio, implies that a decreased content of wood particles to the polymer matrix, biopolymer composites made of *Cordia* *milleni* will have high flexural enhancement. This could be attributed to the nature and percentage of chemical composition found in *Cordia milleni*^37^ reported that the presence of cellulose, hemicelluloses, lignin and other components found in *Cordia milleni* at the right proportions confirmed the wood to be very efficacious for various construction works. This attributes shows that *Cordia milleni* is an ideal raw material for the lignochemical industry which can replace the petrochemical industry for the production of plastic and also aid in the production of all kinds of chemical products for food and textile products^37^. It is possible that this particular chemical component of *Cordia milleni* could have enhanced existed matrix support to wood fiber thereby giving stronger inter-structural bonds that increased the stress transfer and stress concentration in the composites to give higher strength properties and better dimensional stabilities.

## Conclusions

The results of this investigation revealed that mixing proportion and wood species had a substantial impact on the physical and mechanical properties of biopolymer composites. The proportion of PE/wood and wood species significantly affects the rate of water movement in biopolymer composites and can also alter the mechanical properties of the biopolymer composites. It was revealed that biopolymer composites made from lowed content of plastic to wood content had the best mechanical properties in terms of load capacity bearing, and stiffness and still maintained high dimensional stability. Therefore, it is concluded in this study that biopolymer composites made from 60/40 (PE/wood) performed best in utilization properties for the production of products like floor and wall tiles. Furthermore, among all the wood species investigated, *Cordia* *milleni* was seen to be the best wood among others that can be used for biopolymer products for outdoor, indoor, and load-bearing applications.

## Recommendations

The outcome of this research investigation gave the following recommendations;

- *Cordia milleni* particles could also serve as raw material for the production of biopolymer products for outside and inside applications
- The proportional ratio of PE/wood plays a crucial role in the determination of properties for utilization. All the biopolymer composites made from all the ratios had very low dimensional values. Still, composites from 80/20 show the best outstanding performance in dimensional stability.
- Higher density wood species like *Nauclea diderichii* and *Tectona grandis* show improved strength performance at 80/20 than the medium and low-density wood species (*Cordia* *milleni* and *Gmelina Arborea*).
- In terms of load-bearing capacity, *Cordia* *milleni* particle can be used for the production of biopolymer products for indoor and outdoor applications. In contrast, *Gmelina Arborea*, *Nauclea diderichii*, and *Tectona grandis* can be used in a moist environment.
- More research should be done on various indigenous wood and non-wood species as raw materials to manufacture biopolymer composites products. Furthermore, their chemical composition should also be carried out, as this can also be an influencing factor on the performance of these wood species.
