# Author Correction: Dimensional stability and mechanical properties of extruded-compression biopolymer composites made from selected Nigerian grown wood species at varying proportions

**DOI:** 10.1038/s41598-023-45895-6

**Published:** 2023-11-01

**Authors:** A. O. Oladimeji, F. Z. Agboola, D. O. Oguntayo, K. S. Aina

**Affiliations:** 1 Natural Products Research Laboratory, Industrial Chemistry Unit, Department of Chemical Sciences, Olusegun Agagu University of Science and Technology, Okitipupa, Ondo State Nigeria; 2 Department of Environmental Science and Natural Resources, National Open University of Nigeria, Ibadan Study Center, Ijokodo, Ibadan, Ondo State Nigeria; 3 Department of Civil Engineering, Landmark University, Omu‑Aran, Nigeria; 4 Department of Forest Products Development and Utilization, Forestry Research Institute of Nigeria, Forest Hill, Jericho, Ibadan, P.M.B 5054 Ondo State Nigeria

Correction to: *Scientific Reports* 10.1038/s41598-022-14691-z, published online 22 June 2022

The original version of this Article contained an error in the order of the author names, which was incorrectly given as K. S. Aina, A. O. Oladimeji, F. Z. Agboola and D. O. Oguntayo.

As a result, the affiliation order was incorrect. Additionally, the previous Affiliation 1 was incorrectly given as ‘Department of Forest Products Development and Utilization, Forestry Research Institute of Nigeria, Forest hill, Jericho, P.M.B 5054, Ibadan, Oyo State, USA.' and the previous Affiliation 2 was incorrectly given as ‘Department of Chemistry, Olusegun Agagu University of Science and Technology, Okitipupa, Ondo State, Nigeria.'

The correct affiliations are listed below:

1. Natural Products Research Laboratory, Industrial Chemistry Unit, Department of Chemical Sciences, Olusegun Agagu University of Science and Technology, Okitipupa, Ondo State, Nigeria.
2. Department of Environmental Science and Natural Resources, National Open University of Nigeria, Ibadan Study Center, Ijokodo, Ibadan, Oyo State, Nigeria.
3. Department of Civil Engineering, Landmark University, Omu-Aran, Nigeria.
4. Department of Forest Products Development and Utilization, Forestry Research Institute of Nigeria, Forest hill, Jericho, P.M.B 5054, Ibadan, Oyo State, Nigeria.

The original Article has been corrected.

